# Serological Evidence of Natural Exposure to Tick-Borne Pathogens in Horses, Romania

**DOI:** 10.3390/microorganisms9020373

**Published:** 2021-02-12

**Authors:** Andreea Monica Bogdan, Mariana Ionita, Ioan Liviu Mitrea

**Affiliations:** Department of Parasitology and Parasitic Diseases & Animal Biology, Faculty of Veterinary Medicine, University of Agronomic Sciences and Veterinary Medicine of Bucharest, 11464 Bucharest, Romania; andreeabogdan01@yahoo.com (A.M.B.); liviumitrea@yahoo.com (I.L.M.)

**Keywords:** tick-borne pathogens, horses, Romania, serological test

## Abstract

The purpose of this study was to investigate the seroprevalence of selected tick-borne-pathogens (TBPs) among Romanian horses. For this, a total of 223 animals originating from north, central, and southeast Romania, including horses from stud farms (*n* = 118) and working horses (*n* = 105), were tested using a commercial rapid ELISA-based test. Overall, 10.3% (95% confidence interval (CI): 6.7–15.1%) of the tested horses were seropositive for antibodies (Ab) against *Anaplasma phagocytophilum.* Additionally, 18.8% (95% CI: 13.9–24.6%) and 0.5% (95% CI: 0.01–2.5%) of horses were seropositive for Ab against *Borrelia burgdorferi* sensu lato and *Ehrlichia* spp., respectively. Among the tested horses, 3.1% were seroreactive to two or three pathogens. These findings show the natural exposure of Romanian horses to zoonotic tick-borne pathogens and emphasize the need for further studies to better understand the epidemiology of equine tick-borne diseases in Romania.

## 1. Introduction

Ticks are recognized among the main vectors of diseases in humans and animals, due to their wide distribution and the wide variety of pathogens they transmit, including pathogens with zoonotic potential. In recent years, their importance has increased all over the world, since the (re)-emergence and spread of tick-borne diseases (TBD) have become a threat to public and animal health [1,2]. Among the tick-borne pathogens (TBPs), *Anaplasma phagocytophilum*, Lyme disease-associated *Borrelia* spp., and several *Rickettsia* spp. are the most common pathogens reported to infect humans and animals, including horses [3,4].

The zoonotic tick-borne pathogens *A. phagocytophilum* and *B. burgdorferi* s.l. are on the rise [3,5], but little is known about their distribution in horses.

*A. phagocytophilum* (order: Rickettsiales, family: Anaplasmataceae) is a widespread, multihost, obligate intracellular Gram-negative bacterium that is able to infect the granulocytes, mainly neutrophils, of several wild and domestic animal species, including horses. It is the causative agent of tick-borne fever of ruminants (TBF) and of human, canine, feline, and equine granulocytic anaplasmosis (HGA, CGA, FGA, and EGA, respectively) [3,6]. *A. phagocytophilum* is also regarded as an emerging zoonotic pathogen which is becoming increasingly recognized in the Northern Hemisphere [2,7].

Horses infected by *A. phagocytophilum* develop a disease known as equine granulocytic anaplasmosis (EGA) (formerly equine granulocytic ehrlichiosis), characterized by fever, depression, anorexia, ataxia and reluctance to move, limb edemas, icterus, and petechiae; laboratory pathology may include anemia, leukopenia, and thrombocytopenia [8,9,10]. Additionally, neurological signs have recently been reported [11].

For all species, *A. phagocytophilum* is transmitted through the bite of an infected tick. In Europe, *Ixodes ricinus* is the main vector, while a variety of wild mammals, such as wild ruminants, rodents, and insectivores may represent efficient reservoir hosts, contributing to endemic cycles [3]. Transstadial transmission is considered important in maintaining of *A. phagocytophilum* within enzootic cycles, as transovarial transmission has not described for this tick. Recently, four different *A. phagocytophilum* ecotypes (genetic variants) were identified which could circulate within different enzootic cycles in relation to species of vertebrate host and tick vector. Of these, ecotype I has the highest zoonotic potential and the broadest host range, including horses [12].

*B. burgdorferi* s.l. is the causative agent of Lyme disease, an important tick-borne disease in humans and various animals, including horses [13]. Most *B. burgdorferi* s.l.-infected horses are asymptomatic, but there are some documented naturally occurring syndromes associated with *B. burgdorferi* infection which include neuroborreliosis, cutaneous pseudo-lymphoma, and uveitis [14].

Studies on the presence and distribution of tick-borne pathogens in Romanian horses are very scarce. Several previous studies in Romania indicated the circulation of some TBPs at the tick–host interface, demonstrated by molecular investigations. In particular, DNA of *A. phagocytophilum* has been detected in *I. ricinus*, both feeding ticks collected from cattle and horses [15] and questing ticks [16], and tissue samples collected from red foxes and rodents [17,18], while *B. burgdorferi* s.l. DNA has been detected in questing ticks [19] and feeding ticks collected from horses [15]. However, there is very scant information about the spread of these pathogens among horses reared in Romania. Therefore, the aim of the study was to investigate the infection status of *A. phagocytophilum* and other selected zoonotic tick-borne pathogens in equine populations from different geographical regions of Romania and to evaluate a possible role of horses in the epidemiology of zoonotic tick-borne diseases.

## 2. Materials and Methods

### 2.1. Study Areas, Animals, and Investigations

**Figure 1:**
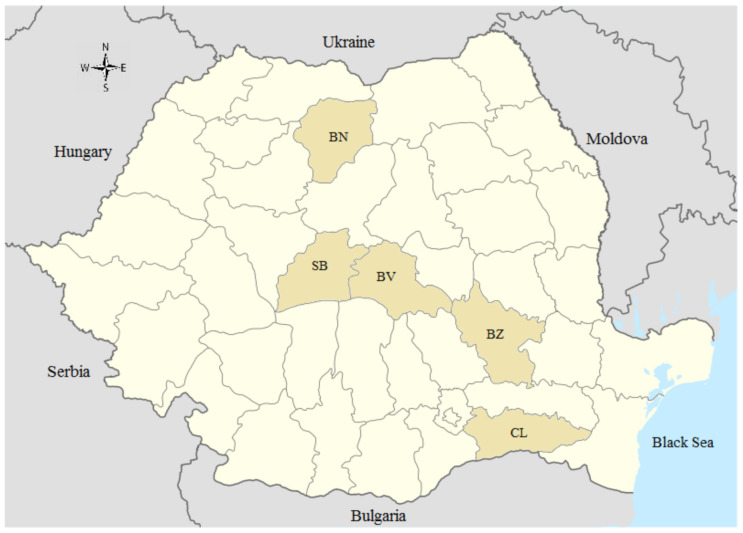
Map of Romania showing counties where horse sampling was carried-out. BN—Bistrita Nasaud; SB—Sibiu; BV—Brasov; BZ—Buzau; CL—Calarasi.

Between June 2017 and October 2019, a total of 223 horses originating from five counties (administrative units) in north, central, and southeast Romania were included in the study (Figure 1).

Horses were actively racing and reared in stud farms and in small individual farms (working horses) located in hilly and lowland areas of Romania.

Details related to age, gender, health status, breed, use, and origin were registered for each animal.

According to their activities, animals were categorized into two groups on the basis of raising system: stud farms and working horses.

Horse blood samples were collected from the jugular vein in ethylenediaminetetraacetic acid (EDTA)-sterile blood collection tubes.

All samples were tested for the presence of circulating antibodies (Ab) against *A. phagocytophilum*, *B. burgdorferi* sensu lato, and *Ehrlichia* spp. using a commercially available ELISA-based test (SNAP^®^ 4Dx^®^ Plus; IDEXX Laboratories, Inc.), according to the manufacturer’s instructions.

The SNAP^®^ 4Dx^®^ assay was originally developed to detect Ab against *A. phagocytophilum*, *B. burgdorferi* s.l., and *Ehrlichia* spp. (*E. canis, E. chaffeensis*, and *E. ewingii*) in dogs but it has also shown useful results in horses [20,21,22]. The assay uses an all-species conjugate and it is not species-specific for animals; moreover, it has been validated for horses, with 100% sensitivity and specificity for the detection of antibodies against *A. phagocytophilum* and 100% and 95%, respectively, for *B. burgdorferi* s.l.; furthermore, no cross-reactions with either *E. canis* or the heartworm analytes in the SNAP^®^ 4Dx^®^ assay were found [23].

### 2.2. Statistical Analysis

The collected data were statistically analyzed using Quantitative Parasitology 3.0 software. The prevalence and corresponding 95% confidence intervals (95% CI) were calculated. Differences among categories were assessed by means of Fisher’s exact test and were considered statistically significant for values with *p* < 0.05.

## 3. Results

To assess the infection status of *A. phagocytophilum* and other selected tick-borne pathogens, namely, *B. burgdorferi* s.l. and *Ehrlichia* spp., in horses reared in Romania, a total of 223 animals, including 118 horses from stud farms and 105 working horses, originating from three geographical regions, were serologically tested.

Details related to the geographical characterization of the horses’ originating areas are presented in Table 1.

Horses aged between 8 months and 26 years (mean 9 years; standard deviation (SD) 5.4) were of both genders and pure- and mixed-breed (Table 2).

Horses were used for different activities. Horses from stud farms were used for breeding, races, and leisure, while working horses, which are raised in small, individual farms in rural areas of Romania, were used for various outdoor field activities, such as harvesting and transport of crops, working agricultural land, and forestry work.

All animals were apparently clinically healthy at the time of blood withdrawal and past or existing tick-borne diseases were not registered for any of them.

Altogether, 26.9% (95% CI: 21.2–33.2%) of the tested animals were positive for at least one of the tested pathogens.

Out of 223 horses, 23 (10.3%; 95% CI: 6.7–15.1%) were seropositive for Ab against *A. phagocytophilum*, whereas 42 (18.8%, 95% CI: 13.92–24.6%) and one (0.5%, 95% CI: 0.01–2.5%) were seropositive for Ab against *B. burgdorferi* s.l., and *Ehrlichia* spp., respectively.

Positive animals for *A. phagocytophilum* and *B. burgdorferi* s.l. were identified in all three regions represented in this study. The mean frequency by region varied from 5.0% to 11.7% for *A. phagocytophilum*, while that for *B. burgdorferi* s.l. varied from 7.5% to 23.8%. Higher seroprevalence values were registered in working horses than those from stud farms: 14.3% versus 6.8% for *A. phagocytophilum*; 28.6% versus 10.2% for *B. burgdorferi* s.l.

One male, mixed-breed working horse originating from central Romania was seroreactive for *Ehrlichia* spp. infection.

In 2.7% (6/223) and 0.5% (1/223) of samples, mixed infections with *A. phagocytophilum* plus *B. burgdorferi* s.l. and with *A. phagocytophilum* plus *B. burgdorferi* s.l. plus *Ehrlichia* spp., respectively, were detected.

Details on the prevalence of each pathogen by horse category and originating area are shown in Table 2.

## 4. Discussion

Studies on the presence and distribution of tick-borne pathogens in Romanian horses are very scarce. *B. caballi* and *T. equi* infections have been recently reported in asymptomatic horses in southeast Romania [24], while another recent study using molecular evidence also documented a clinical equine babesiosis outbreak caused by *B. caballi* in a tick-endemic area in Southern Romania [25]. Therefore, the present study provides serological evidence on the exposure of Romanian horses to three other tick-borne pathogens, namely, *A. phagocytophilum*, *B. burgdorferi* s.l., and *Ehrlichia* spp., for which little or no data are available.

The seroprevalence of *A. phagocytophilum* (10.3%) in the present study is consistent with some serological surveys conducted in European countries, such as in northern Bulgaria (12.0%) [26] and France (13.5%) [21]. However, higher infection rates were reported in horses from Denmark (22.3%) [20] or southern Bulgaria (20.0%) [27]. All these studies used the same test technology. Other serological surveys used immunofluorescence assay (IFA) and ELISA for the detection of *A. phagocytophilum* in horses, such as in Italy (9.0%; 13.4%; 7.4%) [28,29,30], France (11.3%) [31], Sweden (17.0%) [32], Spain (6.5%) [33], and Portugal (3.0%) [34].

Similarly, quite wide ranges of prevalence values have also been reported from PCR-based surveys, from 0.33% to 25.62% [3,9,34].

Furthermore, our result showed higher seroprevalence values in working horses than horses raised in stud farms (14.3% versus 6.8%). This could be explained by the fact that working horses, being used in various outdoor field activities, are more exposed to ticks. There were no statistically significant differences in the seroprevalence according to the any horse category. However, older horses (>10 years) showed higher *A. phagocytophilum* seroprevalence (13.0%) than younger horses, which could be explained by the fact that older horses are more likely to have been exposed to vector ticks.

To the best of our knowledge, this is the first serological survey on the occurrence of *A. phagocytophilum* in horses reared in Romania. However, *A. phagocytophilum* infection was reported in other domestic animal species in Romania, such as dogs, with a serological prevalence ranging from 2.25% to 16.0% [35,36]. The findings of the present study confirm the presence of this zoonotic tick-borne pathogen in horses in the investigated areas and suggest that horses can contribute to the natural cycle of this bacterium in Romania. Moreover, as all the seropositive horses were apparently clinically healthy, these findings support previous studies indicating that up to 50% of seropositive horses could respond immunologically to *A. phagocytophilum* exposure without developing clinical signs. Additionally, clinical anaplasmosis is very likely to be underdiagnosed given that most horses recover spontaneously and clinical signs are not specific, being rather similar to other infections [9]. Subsequently, clinicians should be informed about this fact, so that they can evaluate clinical disease, especially for horses newly introduced into an endemic area, which are more likely to develop illness [3,34].

In addition to *A. phagocytophilum*, two other tick-borne pathogens, *B. burgdorferi* s.l. and *Ehrlichia* spp., respectively, were serologically detected in horses in the investigated areas. Of those, *A. phagocytophilum* and *B. burgdorferi* s.l. are both transmitted by *I. ricinus* ticks.

In our study, Ab against *B. burgdorferi* s.l. were detected in horses originating from all three regions, with a mean frequency of 18.8%, ranging from 7.5% to 23.8%, by region. The findings show that most of the horses seroreactive for *B. burgdorferi* s.l. were found in central (23.8%) and southeast (18.4%) regions, thus suggesting a possibly higher tick density and a higher risk of becoming infested with an infected tick. This is also supported by a molecular study that reported higher infection rates (22.0% and 19.0%) of questing *I. ricinus* ticks in central (Sibiu county) and southeast (Giurgiu county) Romania [19]. These reports provide evidence for the presence of a causative agent for Lyme disease in ticks from various regions of Romania, posing risks for human and animal infections in these areas.

In addition to this, a previous Romanian study, which to the best of our knowledge is the only study at this moment, on the seroprevalence of *B. burgdorferi* s.l. in horses showed a global seroprevalence of 11.92%, but no correlations were found with age, gender, county, or occupation [37]. In our study, seroreactivity was correlated with raising system, gender, and breed, with male, mixed-breed, and working horses showing higher infection rates. This suggests that these horses, being often used outdoor for field duties, are more exposed to ticks and vectored pathogens. Additionally, local regional ecological factors may have an impact on the infection rate favoring the natural circulation of *B. burgdorferi* s.1. [13,14].

The *B. burgdorferi* s.l. seroprevalence of 18.8% in our study is consistent with other European surveys, such as in Bulgaria (15.5%; 23.2%) [26,27], Sweden (16.8%) [38], and Germany (16.1%) [39]. Higher infection rates were reported for horses in Denmark (29.0%) [20], Poland (25.6%) [40], and Slovakia (47.8%) [41]. Furthermore, 4.5%, 6.3–7.1%, and 11.0% of the examined horses in Sweden, Bulgaria, and Denmark, respectively, were seropositive for both *B. burgdorferi* s.l. and *A. phagocytophilum* [20,26,27,38]. The presence of dual infections is not surprising since both pathogens are vectored by *I. ricinus* ticks; however, coinfection with *A. phagocytophilum* and *B. burgdorferi* s.l. can increase disease severity, as recently reported [4]. In our study, 2.7% of the tested horses were co-exposed to *A. phagocytophilum* and *B. burgdorferi*, suggesting the possible association of *A. phagocytophilum* with *B. burgdorferi* s.l. in terms of endemic patterns and foci. Therefore, horses might be used as sentinels for early detection of emerging tick-borne diseases.

Several recent studies from North America (South Central United States, Oklahoma), South America (Brazil), and Central America (Nicaragua) documented reactivity to *Ehrlichia* spp. in horse serum [42,43], and molecular and phylogenetical studies suggest a potentially novel *Ehrlichia* species different from currently recognized species [22,44]. However, the main vector and the clinic significance of this newly identified equine *Ehrlichia* species are yet unknown.

In Europe, two recent serologic studies from Bulgaria, a neighboring country, also reported the seroreactivity to *Ehrlichia* spp. in horses (seroprevalence values of 0.5% and 3.9%, respectively) [26,27]. In our study, one horse (0.5%) was seroreactive to *Ehrlichia* spp., and it was also positive for *A. phagocytophilum* and *B. burgdorferi* s.l. A previous study in Romania confirmed the presence of *E. canis* DNA in ticks infesting dogs, in southeast Romania [45]. However, future extended surveys are planned to provide additional data for understanding if Romanian horses are reactive to *E. canis* or to a potential novel *Ehrlichia* species, as recently described [44].

Therefore, this preliminary work opens new avenues for further serological and molecular research to investigate the infection status of *Ehrlichia* spp. and other tick-borne pathogens in horse populations in Romania, for a better understanding of the epidemiology of equine tick-borne diseases.

## Figures and Tables

**Table 1:** Geographic characterization of the horses’ originating areas.

Region/ County	Relief [Altitudes]	Climate [T*]	Vegetations
North			
BN	Lowlands and hills (350–800 m)	Continental (8–9 °C)	Meadows and pastures that alternate with forests
Center			
BV	Sub-mountain hills (500–800 m)	Continental (8–9 °C)	Meadows and pastures that alternate with forests
SB	Lowlands and hills (400–500 m)	Continental (8–9 °C)	Meadows and pastures that alternate with forests
Southeast			
BZ	Lowlands and hills (80–300 m)	Continental (10–11 °C)	Meadows and pastures that alternate with forests
CL	Lowlands (15–75 m)	Continental (10.5–12.5 °C)	Pastures, clusters of shrubs, and forests

BN—Bistrita Nasaud; SB—Sibiu; BV—Brasov; BZ—Buzau; CL—Calarasi; T*—annual average temperature.

**Table 2:** Seroprevalence of *Anaplasma phagocytophilum* (*A. ph.*)*, Borrelia burgdorferi* senso lato (*B. b. s.l.*), and *Ehrlichia* spp. (*Eh.*) in horse populations, Romania (stratified by raising system, age, gender, breed, and region).

Group	Total Tested (*n*)	Total Positive (*%*)		*A. ph.* Positive		*B. b. s.l. Positive*		*Eh.* Positive		*A. ph.* + *B. b. s.l.*		*A. ph.* + *B. b.s.l. + Eh.*	
		*n*	% (95% CI)	*n*	% (95%CI)	*n*	% (95% CI)	*n*	% (95% CI)	*n*	% (95% CI)	*n*	% (95% CI)
*Raising system*			*				*						
stud farm	118	19	16.1 (10.0–24.0)	8	6.8 (2.9–12.9)	12	10.2 (5.4–17.1)	0	0.0 (0.0–3.1)	1	0.8 (0.0–4.6)	0	0.0 (0.0–3.1)
working horses	105	41	39.0 (29.7–49.0)	15	14.3 (8.2–22.4)	30	28.6 (20.2–38.2)	1	1.0 (0.0–5.2)	5	4.8 (1.6–10.8)	1	1.0 (0.0–5.2)
*Age* (years)													
<5	65	12	18.5 (9.9–30.0)	5	7.7 (2.5–17.0)	7	10.8 (4.4–20.9)	0	0.0 (0.0–5.5)	0	0.0 (0.0–5.5)	0	0.0 (0.0–5.5)
5–10	81	24	31.2 (21.0–42.7)	8	9.9 (4.4–18.5)	16	19.8 (11.7–30.1)	0	0.0 (0.0–4.5)	2	2.5 (0.3–8.6)	0	0.0 (0.0–4.5)
>10	77	24	29.6 (20.0–40.8)	10	13.0 (6.4–22.6)	19	24.7 (15.5–35.8)	1	1.3 (0.0–7.0)	4	5.2 (1.4–12.8)	1	1.3 (0.0–0.7)
*Gender*			*				*						
male	88	34	38.63 (28.4–49.6)	12	13.6 (7.2–22.6)	26	29.5 (21.6–38.6)	0	0.0 (0.0–4.1)	6	6.8 (2.5–14.2)	0	0.0 (0.0–4.1)
female	135	26	19.25 (13.0–27.0)	11	8.1 (4.1–14.1)	16	11.9 (6.9–18.5)	1	0.7 (0.0–4.1)	0	0.0 (0.0–2.7)	1	0.7 (0.0–4.1)
*Horse breed*			*				*						
Pure-breed	113	19	16.8 (10.4–25.0)	8	7.1 (3.1–13.5)	12	10.6 (5.6–17.8)	0	0.0 (0.0–3.2)	1	0.9 (0.0–4.8)	0	0.0 (0.0–3.2)
Mixed-breed	110	41	37.3 (28.2–47.0)	15	13.6 (7.8–21.5)	30	27.3 (19.2–36.6)	1	0.9 (0.0–5.0)	5	4.5 (1.5–10.3)	1	0.9 (0.0–5.0)
*Region/ county*													
North	40	5	12.5 (4.2–26.8)	2	5.0 (0.6–16.9)	3	7.5 (1.6–20.4)	0	0.0 (0.0–8.8)	0	0.0 (0.0–8.8)	0	0.0 (0.0–8.8)
BN	40	5	12.5	2	5.0	3	7.5	0	0.0		0.0	0	0.0
Central	80	26	32.5 (22.4–43.9)	9	11.3 (5.3–20.3)	19	23.8 (14.9–34.5)	1	1.3 (0.0–6.7)	3	3.8 (0.8–10.6)	1	1.3 (0.0–6.7)
BV	40	15	37.5	5	12.5	10	25.0	0	0.0	2	5.0	0	0.0
SB	40	11	27.5	4	10.0	9	22.5	1	2.5	1	2.5	1	2.5
Southeast	103	29	28.2 (19.7–37.8)	12	11.7 (6.2–19.4)	20	19.4 (12.3–28.4)	0	0.0 (0.0–3.5)	3	2.9 (0.6–8.3)	0	0.0 (0.0–3.5)
BZ	58	23	39.7	10	17.2	16	27.6	0	0.0	3	5.2	0	0.0
CL	45	6	13.3	2	4.4	4	8.9	0	0.0	0	0.0	0	0.0
Total	223	60	26.9 (21.2–33.2)	23	10.3 (6.7–15.1)	42	18.8 (13.92–24.6)	1	0.5 (0.01–2.5)	6	2.7 (1.0–5.8)	1	0.5 (0.01–2.5)

* Groups for which *p* < 0.05.
